# Low Temperature Annealed Zinc Oxide Nanostructured Thin Film-Based Transducers: Characterization for Sensing Applications

**DOI:** 10.1371/journal.pone.0132755

**Published:** 2015-07-13

**Authors:** R. Haarindraprasad, U. Hashim, Subash C. B. Gopinath, Mohd Kashif, P. Veeradasan, S. R. Balakrishnan, K. L. Foo, P. Poopalan

**Affiliations:** 1 Biomedical Nano Diagnostics Research Group, Institute of Nano Electronic Engineering (INEE), Kangar, Perlis, Malaysia; 2 School of Bioprocess Engineering, University Malaysia Perlis (UniMAP), Kangar, Perlis, Malaysia; 3 School of Microelectronic Engineering, University Malaysia Perlis (UniMAP), Kuala Perlis, Perlis, Malaysia; Institute for Materials Science, GERMANY

## Abstract

The performance of sensing surfaces highly relies on nanostructures to enhance their sensitivity and specificity. Herein, nanostructured zinc oxide (ZnO) thin films of various thicknesses were coated on glass and p-type silicon substrates using a sol-gel spin-coating technique. The deposited films were characterized for morphological, structural, and optoelectronic properties by high-resolution measurements. X-ray diffraction analyses revealed that the deposited films have a *c*-axis orientation and display peaks that refer to ZnO, which exhibits a hexagonal structure with a preferable plane orientation (002). The thicknesses of ZnO thin films prepared using 1, 3, 5, and 7 cycles were measured to be 40, 60, 100, and 200 nm, respectively. The increment in grain size of the thin film from 21 to 52 nm was noticed, when its thickness was increased from 40 to 200 nm, whereas the band gap value decreased from 3.282 to 3.268 eV. Band gap value of ZnO thin film with thickness of 200 nm at pH ranging from 2 to 10 reduces from 3.263eV to 3.200 eV. Furthermore, to evaluate the transducing capacity of the ZnO nanostructure, the refractive index, optoelectric constant, and bulk modulus were analyzed and correlated. The highest thickness (200 nm) of ZnO film, embedded with an interdigitated electrode that behaves as a pH-sensing electrode, could sense pH variations in the range of 2-10. It showed a highly sensitive response of 444 μAmM^-1^cm^-2^ with a linear regression of R^2^ =0.9304. The measured sensitivity of the developed device for pH per unit is 3.72μA/pH.

## Introduction

Sensitivity is the prime determining factor of the quality of sensors, and the construction of nanostructure on the sensing surface creates an avenue for high-performance sensing [1]. Among different sensors proposed, pH sensor has been considered as mandatory to maintain the quality of food by measuring the acidity levels, which will prevent foodborne pathogens. Previously, pH sensors have been fabricated via various types of electrode system by implementing conventional strategy. In order to overcome some limitations, such as rigid and bulk size structure of glass electrode, researchers developed smaller and robust electrode systems, which includes Ion-Sensitive Field- Effect transistor (ISFET) and extended-gate field-effect-transistor (EGFET) [2,3]. However, the complexity of these non-interdigitated electrode device fabrications as well as electrical operations increases the cost, due to the usage of multiple mask sets, which greatly increases fabrication processes. Furthermore, ZnO integration encounters the problems with conventional CMOS processes and possible contamination of process equipment. Additionally, these three terminal devices require complex biasing arrangements in comparison to capacitive interdigitated electrode (IDE). An attempt has been made to develop IDE for pH sensing application since IDE has become promising for developing biosensor system due to its higher stability, robust and simplicity. In addition, ultra-low volume of solution is required for measurements in IDE compared to other electrode systems such as glass electrode, ISFET and EGFET.

Zinc oxide (ZnO) has become a popular choice for the development of sensing platforms with various applications, such as photovoltaics, solar cells, and optoelectronic biosensors [4–7] due to its appealing characteristics such as a large band gap of 3.37 eV [8] and biocompatibility [9,10]. Intensive reviews indicate that ZnO has become an emerging contender for environmental applications with the considerations of reduced cost, non-toxicity, and high reactivity. Studies that focused on the morphological aspects of ZnO materials prepared by the sol-gel method have led to novel applications. ZnO have been attested by researchers to be an ideal material for making nanostructures, due to their ease of synthesis, resulting in a high crystallinity with a few structural defects using a low-temperature process. ZnO also possesses excellent electrical characteristics that can be tailored for a fast and accurate sensor response [11,12]. The biocompatibility of ZnO makes it convenient for surface modification and interfacing with chemical and biological compounds at extreme pH conditions.

The ZnO seed solution, which is indexed to the wurtzite structure associated with high crystallinity, has a high tendency to synthesize quasi one-dimensional nanowires via bottom–up approaches, such as the vapor-liquid-solid mechanism, pulsed laser, flame transfer synthesis, RF magnetron sputtering and hydrothermal methods [13–17]. Prior to the formation of ZnO nanostructures, studies on the crystallographic and morphological properties of ZnO thin films are necessary to analyze the various parameters of the seed solution that affect the thin film formation.

The parameters relevant to the synthesis of ZnO thin films include the thickness, pre-annealing and post-annealing temperatures maintained during processing, sol-seed concentration, and annealing period. In this study, a ZnO seed solution is synthesized via the sol-gel method, and the thickness of the seed solution is tuned to investigate the optoelectronic and structural properties of ZnO thin films that are coated on glass and silicon substrates. Furthermore, we characterized the optical and bulk modulus properties with respect to thickness to evaluate the transducing capacity. The effect of optical properties of ZnO thin film (200 nm) at different pH conditions have been characterized by measuring the bandgap of the membranous ZnO thin film. We made an approach of developing an optimized ZnO thickness, for two electrode detection scheme (IDE) with simple current detection. With a view for high-performance sensing applications, the electrical behaviors on ZnO thin films of different thicknesses under varied pHs from 2 to 10 were investigated.

## Materials and Methods

### Consumable Materials

ZnO seed solution was prepared using zinc acetate dehydrate [Zn(CH3COO)_2_.2H_2_O] (98%; Sigma-Aldrich) as a precursor. Isopropyl alcohol (IPA; 99.8%) was from Sigma-Aldrich. Monoethanolamine (MEA; 99%; Merck) was used as a stabilizer. Silicon wafers (100) were purchased from Filmetrics, and glass slides were purchased from Fisher Scientific.

### Silver Interdigitated electrode (IDE)

A silver IDE electrode was deposited on the silicon wafer <100> using the traditional wet etching method. A positive photo resist (PR) was coated on the silicon wafer followed by soft baking for 90 sec. UV light was applied for 10 sec to pattern transfer the IDE mask. The development took 15 sec using RD-6 developer, and the sample was baked at 110°C to remove excess moisture and enhance the adhesion force between the silver and SiO_2_ layers. The unexposed area was removed by silver etchant applied for 23 sec, and the sample was then cleaned with acetone.

### ZnO synthesis

The ZnO seed solution was synthesized as previously reported [18–20]. Briefly, 1632 mg of zinc acetate powder was diluted in 40 ml of isopropyl alcohol to obtain 0.2 M ZnO seed solution. The seed solution was then stirred at 1000 rpm for 20 min at 60°C. The volumes of the sol stabilizer and monoethanolamine (MEA) were measured to ensure that the ratio between the solvent and stabilizer was 1:1 since F. Boudjouan *et al*. has stated that the most optimum ratio of MEA solvent is 1:1 for synthesizing ZnO sol gel solution [21]. After stirring the solvent for 20 min, the MEA was subsequently added for 120 min until a clear and transparent solution appeared. The prepared ZnO seed solution was left to age at room temperature for 24 h.

### ZnO seeding

The ZnO seed solution was coated on p-type silicon and glass substrates of dimensions 2 x 2 cm^2^. Both substrate types underwent an initial standard cleaning process prior to coating with the ZnO seed solution. The prepared ZnO seed solution was coated via the spin-coating method. The coated samples were heated at 60°C for 20 min, at point where the temperature was raised to 150°C and kept constant for 10 min before cooling to 50°C. The samples were heated after each coating to eliminate residual moisture and enhance the adhesion between the surface of the substrate and the ZnO seed solution. After the coating, all samples underwent an annealing process at 300°C for 2 h.

### Characterization of nanostructured ZnO thin films

The morphological characterization of the nanostructured ZnO thin films was conducted using field emission scanning electron microscopy (FESEM; Carl Zeiss AG-ULTRA 55, Gemini). The structural analysis was conducted via X-ray diffraction (XRD, Bruker D2 Phaser). Atomic force microscopy (AFM; SPA400-SPI3800) was utilized to examine the roughness of the film surfaces. The optical properties were examined by ultraviolet-visible spectroscopy (UV-vis, Lambda 35, Perkin Elmer), and photoluminescence (PL) spectroscopy (PL, Horiba Fluorolog-3, HORIBA Jobin Yvon Inc; USA) was used to observe the effect of the thickness on the optical transmittance and crystal defects. The electrical characterization was conducted using a Keithley 2400 source meter.

## Results and Discussion

### Analysis

A zinc oxide (ZnO) thin film was synthesized by a simple chemical route (sol-gel) and optimized to improve its internal and surface matrixes for sensing applications. The characteristics of the thin film were modified by the deposition of different thicknesses on the surface of the sensing electrode. The structural, morphological, optical and electrical changes upon varying the thicknesses of the ZnO thin film were critically observed.

### Structural analysis

**Fig 1:**
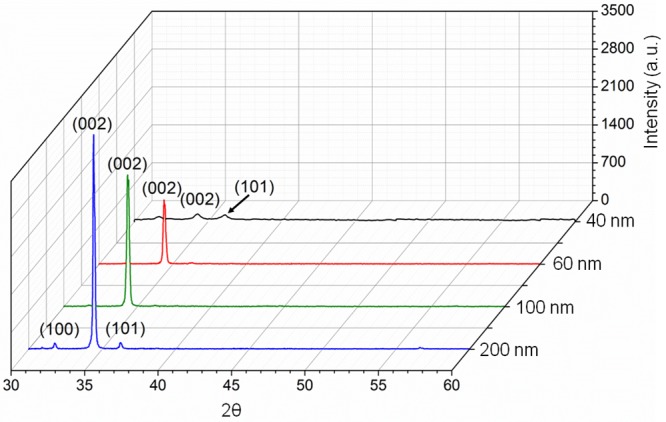
Structural and grain size analysis of matrix of ZnO thin film. X-Ray diffraction analyses on ZnO thin films.

ZnO thin films of different thicknesses were observed by X-ray diffraction (XRD) analysis, and preliminary investigations were evaluated for the grain crystal size, grain growth orientation and crystalline quality of the matrix. Fig 1 displays the XRD patterns of ZnO thin films of different thicknesses. The XRD spectra shows that all films consist of (100), (002), and (101) plane peaks. The presence of these peaks indicate that all deposited films have only ZnO materials that are indexed by a hexagonal wurzite structure, which is in good agreement with the standard diffraction pattern (JCPDS card # 36–1451).

As the film thickness was increased, the intensity of the diffraction peaks [(100), (002), and (101)] were also increased, revealing that the crystallinity was increased in the thin film due to the preferential growth of ZnO along the *c*-axis [22]. The Scherrer equation was used to calculate the crystallite size of the as-prepared films:

$$D=\frac{\kappa \;\lambda }{FWHM\text{cos}\theta }$$ (1)

where, κ is the Scherrer constant, with a value of 0.9; λ represents the incident X-ray wavelength; FWHM is the full-width at half-maximum of the respective peak; and θ represents the diffraction peak angle.

The grain size was increased with the number of coating cycles, as shown in Table 1. The grain size of the seed solution was calculated based on the highest peak orientation. Thus, calculations were conducted on the (002) orientation based on the XRD data. The crystallinity of the ZnO thin film was enhanced upon increasing the film thicknesses, as calculated by the reduction of the FWHM value [23]. The strain percentage [ε (%)] was decreased with increasing film thicknesses, which is mainly due to low temperature (200°C) and longer annealing time. Consequently, it is also suspected that it cause the reduction in RMS surface roughness. An increase in the film thickness leads to reduced surface roughness, which is more suitable for the function of a transducer in optoelectronic applications due to its uniformity. Strain, ε (%) was calculated based on the following equation:

$$\frac{C-C_{o}}{C_{o}}\;x\;100\%$$ (2)

where C is the lattice constant, which was calculated using Bragg’s equation, and Co is the lattice parameter for bulk ZnO, which has a fixed value of 0.5206 nm [24].

**Table 1:** Calculated grain size, full width at half maximum (FWHM), and strain of ZnO films with different thickness layers using Bragg equation.

ZnO thin film thickness (nm)	Grain size (nm)	FWHM (°)	a (Å)	c (Å)	Strain, $\varepsilon \;\left(\%\right)\frac{\text{C-Co}}{\text{Co}}\;\text{x }100$
40	21.00	0.51	3.257	5.641	8.356
60	41.60	0.10	3.251	5.631	8.164
100	43.00	0.08	3.250	5.629	8.125
200	52.00	0.02	3.246	5.622	7.991

### Morphological Analysis

Morphological observations were conducted on coated ZnO thin films of various thicknesses. FESEM characterization was carried out to inspect the grain geometry (shape and size) of the bipolar matrix in the ZnO thin film. The uniformity and surface smoothness of the coated ZnO thin film was also observed by AFM characterization.

**Fig 2:**
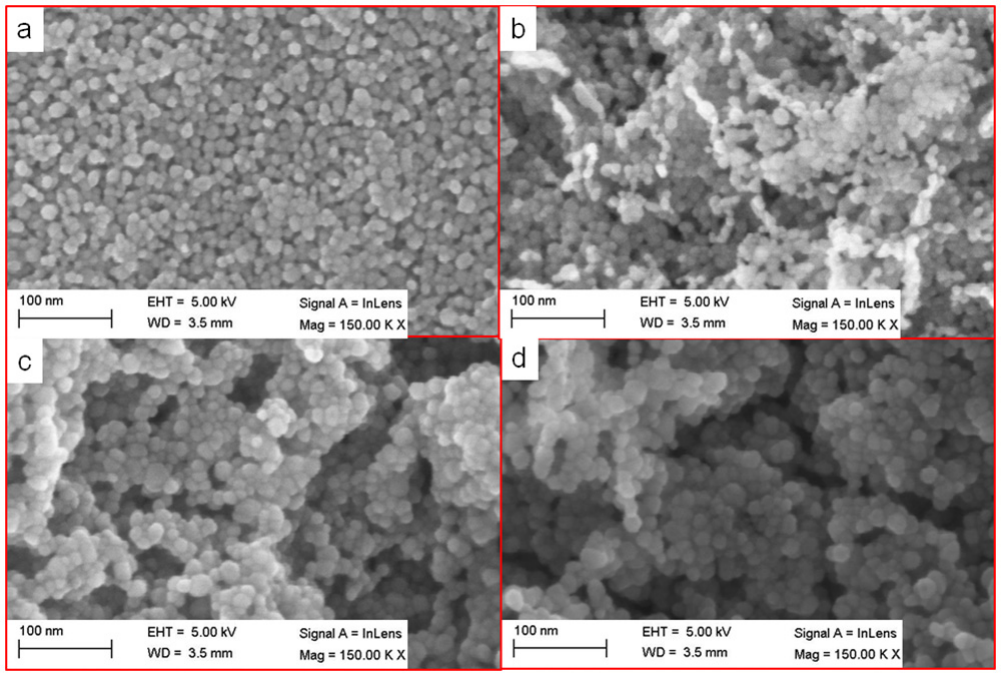
Morphological inspection of ZnO thin films of different thicknesses. Geometrical grain shape analysis and size inspection were conducted to observe the effect of different thicknesses. FESEM images of ZnO thin films coated with different thicknesses (a) 40 nm, (b) 60 nm, (c) 100 nm and (d) 200 nm.

Fig 2 shows FESEM images of ZnO thin films of different thicknesses that were coated on silicon substrates. The average grain size of the ZnO thin film, as observed from the FESEM image, was in the range of 10 to 50 nm. The FESEM images revealed that the thin film surface showed spherule-shaped structures, which is in agreement with the results of Berrin and Sumer [24]. The spherule shape of the seed remains unchanged with additional coating cycles on the glass substrate. However, Aihua et al. reported that the grain shape of the ZnO changed from hexagonal sheet-like to wedge-shaped with the increasing thickness of the thin film [23]. Our results are different mainly because of low temperature and long annealing time were used in the current process. Whereas they used different coating method, and due to the involvement of N-Al doping in the ZnO thin films, there was a change in the shape of the seeded layer. On the contrary, with the current baking-annealing process, the grain size of the seed was enhanced with increasing coating cycles.

The grain was seen as bright-white structure on the lower right corner of Fig 2B, was due to difference degree of contrast in FESEM image. The grain size was increased after undergoing several coating cycles because of the enhanced coalescence between grains of the ZnO thin film. Fig 2A–2D depicts that the FESEM magnification at a constant level (150,000X) for all four film thicknesses produced increasing grain sizes with film thicknesses. This also leads to comparative increment in trap sites [25].

**Fig 3:**
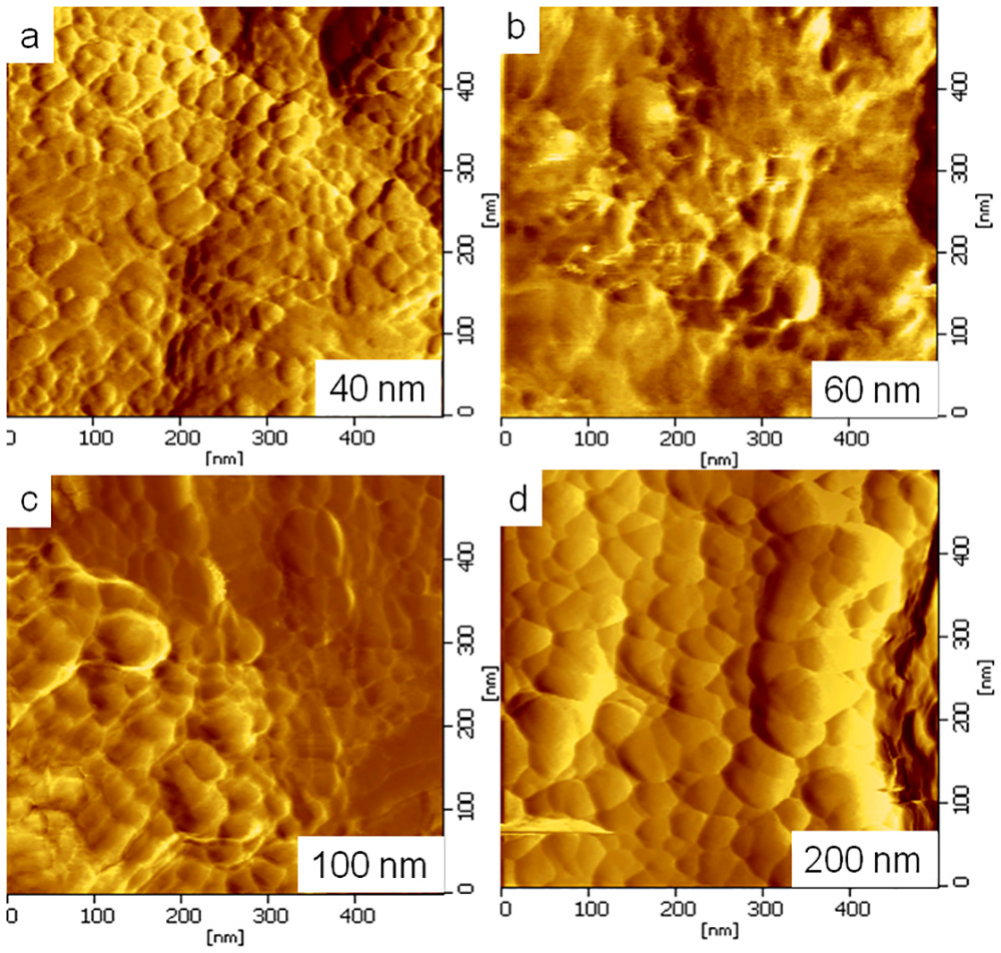
Surface analysis of coated thin film. The surface uniformity and roughness were investigated by AFM. The 2D AFM images of ZnO thin films of different thicknesses are shown.

The uniformity of the thin film was improved with increasing coating cycles, which is in agreement with the AFM result displayed in Fig 3. The reduced surface roughness of the coated thin film is optimal for various applications, such as photovoltaic and solar cells, because of their enhanced ability for photon absorption [26]. Fig 3 shows 2D-AFM images of nanostructured ZnO thin films of different thicknesses. AFM images were captured in tapping mode over an area of 500 x 500 nm at a scanning speed of 2 MHz. Fig 3 also displays that the grain particles of the coated thin film exhibit changes in size and distribution with increasing thicknesses of the thin film. The surface uniformity of the thin film varies non-monotonically. The AFM images showed that the thickness of the thin film and the arrangement of the grain particles strongly affect the surface uniformity of the thin film. The surface uniformity is dominated by the grain particle due to grain coordination. Thus, as reported before, a suitable thin film thickness is needed for further optimization, together with a synthesis of good surface uniformity [27].

**Fig 4:**
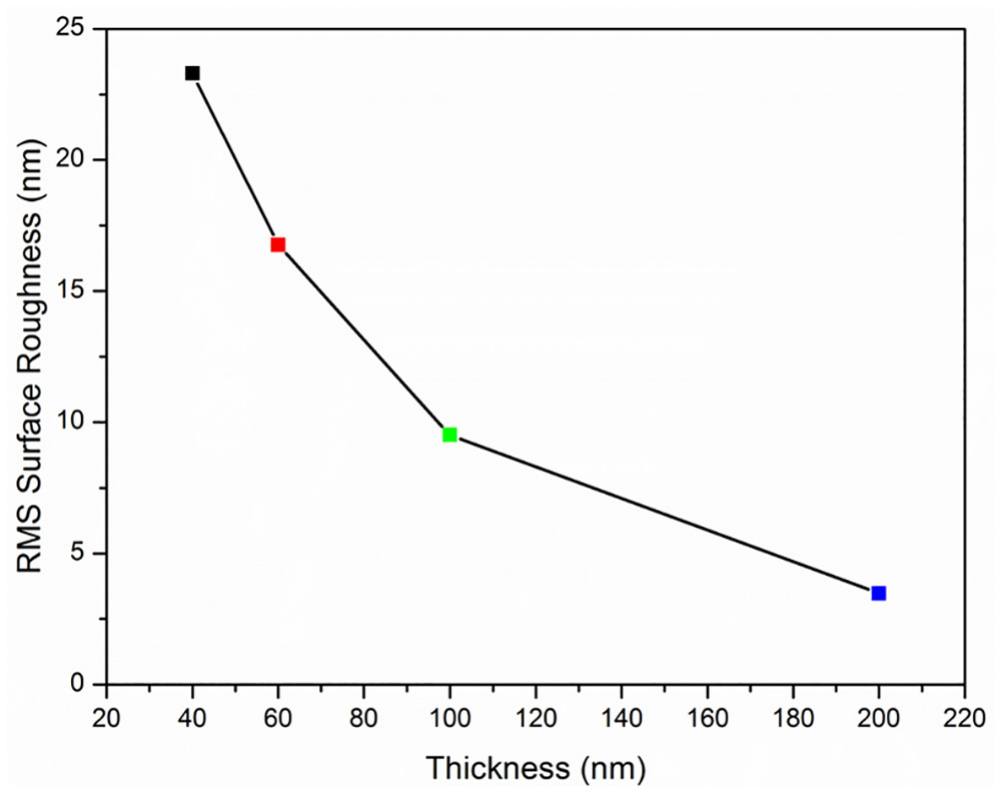
Surface roughness measurement on ZnO thin film. Thickness of ZnO thin film versus RMS surface roughness value.

Fig 4 shows the relationship between the thickness of a ZnO thin film coated on a glass substrate and the root mean square (RMS surface roughness) value of the surface-coated glass substrate. The RMS surface roughness value decreases with increasing thickness of the thin film, indicating that the degree of the surface roughness decreased because of the increment in the grain-size particles of the ZnO [28]. Xu et al. reported similar results regarding the relationship between the thickness of the ZnO thin film and the RMS surface roughness values [29]. Ghayour et al. stated that an increase in the thickness of a ZnO thin film will cause a reduced roughness frequency [30].

**Fig 5:**
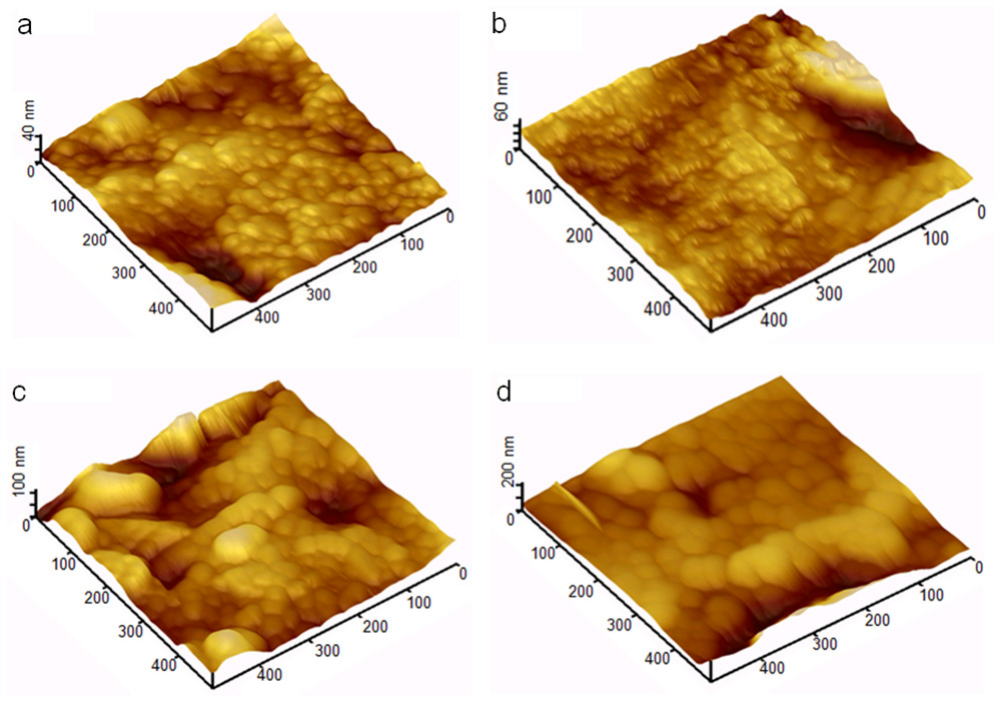
Thickness measurements of thin films undergoing different numbers of coating cycles. 3-D AFM image of surface of glass substrate coated with 1, 3, 5, and 7 coating layers of ZnO seed solution.

Fig 5 shows 3D images of coated ZnO thin films that underwent various coating processes. When the coating cycles on the glass substrate were increased, the thicknesses of the thin film were also increased. Fig 5 also shows large numbers of grain particles at the low thickness layer of the ZnO thin film; thus, the number of grain particles decreased upon increasing the thickness of the thin film. When the number of coating cycles increased from 1 to 7, the ZnO thin film surface uniformity was gradually improved.

### Optical analysis

**Fig 6:**
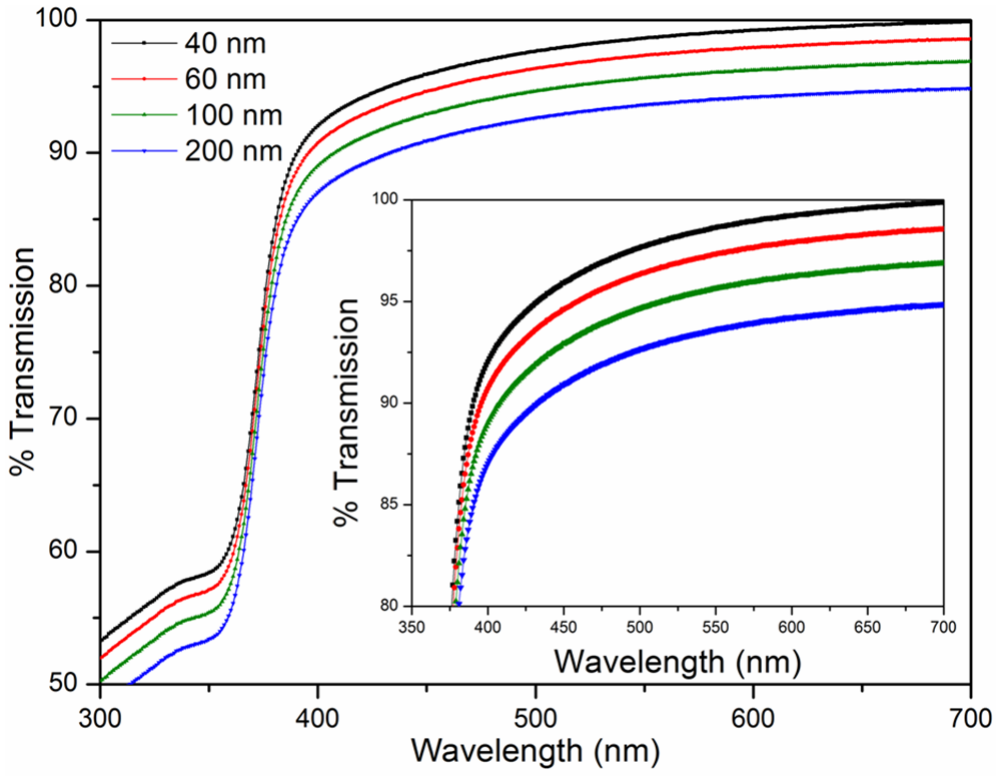
Optical characterization of thin films to evaluate their performances for UV transmission application. Insert shows the magnified scale of transmission spectra of ZnO thin films of various thicknesses.

The prepared ZnO seed solution was coated on glass substrates to investigate the transmission and absorption performances by UV-vis spectroscopy. The quality of the thin film was investigated using photoluminescence (PL) at room temperature. The structural defects in the internal matrix of the ZnO thin film can be identified through PL characterization. Fig 6 shows the effect of thickness on the transmission spectrum of the ZnO thin film. The ZnO thin film showed excellent transmission percentage, which is more than 80%. The transmission percentage of ZnO thin film reduces as the thickness of the thin film increases from 40 to 200 nm. The standard Beer's Law equation explains the reduction of transmittance of ZnO thin film with increasing thickness of ZnO thin film:

$$I=I_{o}e^{-\alpha d}$$ (3)

where α is the absorption co-efficient and d is the film thickness.

The following equation is used to determine the absorption coefficient (*α*):

$$\alpha =\frac{\text{ln}\left(1/T\right)}{d}$$ (4)

where *T* represents transmittance of ZnO films and *d* represents the film thickness of ZnO thin film.

The reduction in the transmission with increasing film thickness may be attributed to the effect on the surface roughness and grain size boundary of the thin films, as described earlier [31–34]. Results obtained from Figs 2 and 3 were validated and depicted that with increasing film thicknesses, the surface roughness and the transmission were reduced. ZnO thin films with a uniform surface exhibited superior absorption capabilities to those with a rough surface.

**Fig 7:**
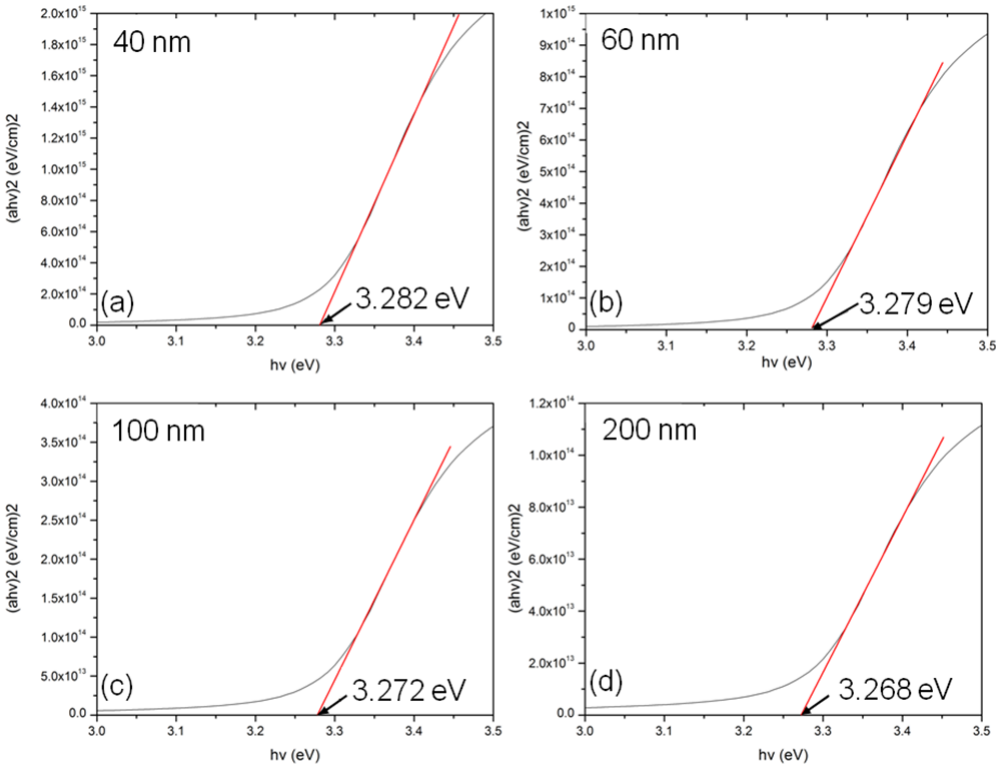
Bandgap observation of coated thin film to investigate the optoelectronic performance. Tauc plot of ZnO thin films of different thicknesses.

Fig 7 shows the bandgap curves of ZnO thin films of different thicknesses. The bandgap was obtained by plotting a Tauc curve, and the values were decreased from 3.282 eV to 3.268 eV as the thicknesses of the ZnO film were increased (40 to 200 nm).

The direct bandgap values of the samples treated with different ZnO thin film thicknesses were obtained by the following formula:

$$\alpha hv=\beta {\left(hv-\text{Eg}\right)}^{n}$$ (5)

where *hv* represents photon energy, β is fixed constant, Eg is the band gap energy, and *n* is the allowed direct band with a value of 1/2.

The decrease in the band gap may be attributed to a reduced deterioration rate of the lattice structure of the ZnO thin film with increasing thicknesses [35]. Previous reports stated that voids in the structure of the thin film could enhance the deterioration rate [36], and these studies have illustrated that the band gap of a ZnO thin film can be altered without introducing foreign dopants.

**Fig 8:**
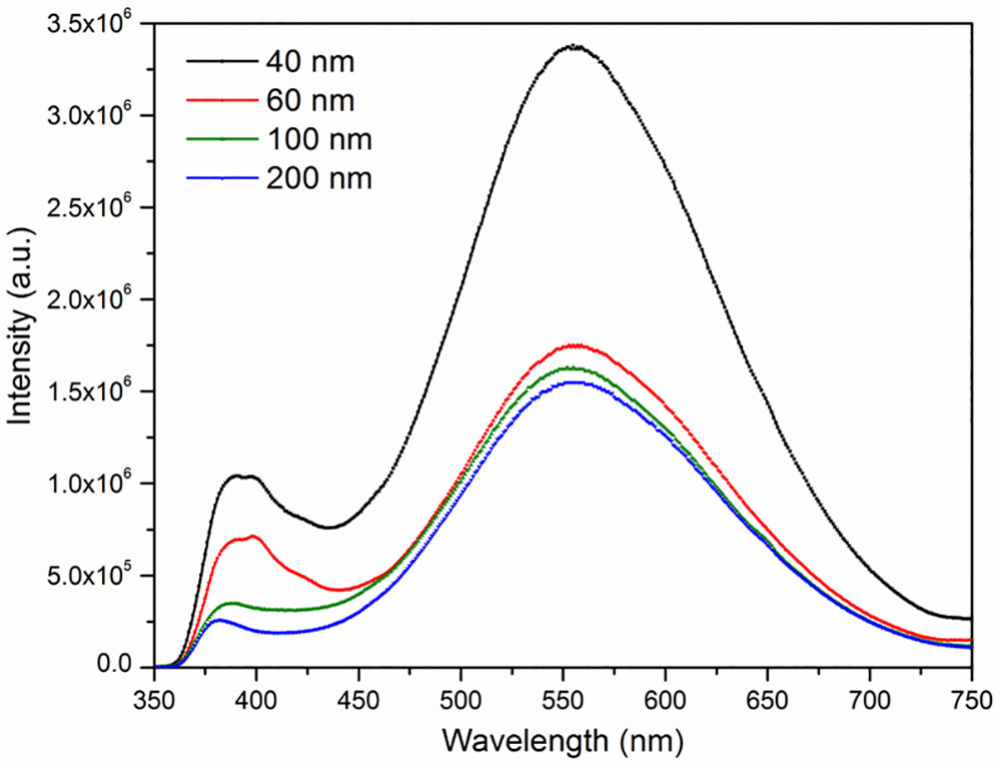
Photoluminescence analysis at room temperature to investigate the effect of thickness on structural quality of ZnO thin film matrix. Photoluminescence spectra of ZnO thin films of different thicknesses are shown.

To further validate the crystalline quality of the thin films, photoluminescence emission spectroscopy was used, and the results are illustrated in Fig 8. PL spectra demonstrated that all the ZnO films exhibited two peaks, one is in the UV range and the other is in the visible range. The small peak in the UV region corresponds to the near band edge, attributed to the radiative recombination of free excitons, and the broad peaks in the visible region are associated with structural defects [37]. Structural defects of a seed solution exhibited in the visible region are referred to as native point defects and include zinc vacancies (V_zn_) and oxygen vacancies (V_o_), which present deep-energy levels of the band gap [38,39]. Voids and imbalances in the Zn-O ratio can cause Zn and O vacancies, leading to structural defects in the thin film [40]. The visible range (from 500 to 700 nm) had a significant decrease in intensity with the increasing thickness of the ZnO thin film, which shows a reduction in the structural defect density in the thin film. This result reveals that increasing the thicknesses of ZnO thin film from 40 to 200 nm, could improve the structural quality, which can contribute its usage as a transducer in biosensing applications.

### Optoelectronic performance analysis

The refractive index ‘n’ is considered a vital physical parameter, and it can be approached from its relationship with the density or the local polarizability [41–46]. The value of ‘n’ is not dependent on temperature or incident photon energy. Ravindra et al. [47] expressed ‘n’ as a linear function of E_g_:

$$\text{n}=\alpha \;+{\beta \text{ E}}_{\text{g}}$$ (6)

where α = 4.048 and β = -0.62 eV^-1^. From the simple physics of light refraction and dispersion, Herve and Vandamme (1995) formulated an empirical relation [48]

$$=\sqrt{1+\;{\left(\frac{\text{A}}{{\text{E}}_{\text{g}}+\text{B}}\right)}^{2}}$$ (7)

Where, A = 13.6 eV and B = 3.4 eV. Ghosh et al. [49] used a different strategy by considering band structure and the quantum dielectric approaches of Penn (1962) [50] and Van Vechten [51]. By indexing ‘A’ as an important contribution of the valence electrons and ‘B’ as a constant additive for the band gap with the lowest E_g_, the high-frequency refractive index can be expressed as:

$${\text{n}}^{2}-1=\frac{\text{A}}{{\left(\text{Eg}+\text{B}\right)}^{2}}$$ (8)

where A = 25E_g_ + 212, B = 0.21E_g_ + 4.25, and (E_g_ + B) refers to the material with an appropriate average energy gap. Verification of the relationship was performed by calculating the dielectric constant *ε*_∞_, in which *ε*_∞_ = *n* ^2^ [52], and it is in agreement with the experimental value [47,53]. The intense absorption and minimal reflection represents an increase in the efficiency of generating optoelectronic devices.

The bulk modulus represents the material stiffness, as observed previously [54–59], and it can be computed accurately by using solid structural and electronic properties. A more empirical derivation was designed with ‘ab’ initio calculations [60]. Cohen [61] formulated an empirical formula to calculate the bulk modulus ‘B0’ based on the nearest-neighbor distance under agreement with the experimental values. Lam et al. [62] designed an analytical expression for the pressure derivative ‘B0’ that is different from the empirical formula in structure, but produces similar numerical results. Douri et al. [63] followed a concept based on the lattice constant and established an empirical formula. A reason to study ‘B0’ is due to the clear differences between the lattice constants of different ZnO samples, as shown in Table 2.

**Table 2:** Energy gaps (eV), lattice constants a (Å), and bulk modulus (GPa) of ZnO films at different current densities, along with theoretical [64,65] and experimental data [66].

Thickness(nm)	a (Å)	B_0_ (GPa)
40	3.257^#^3.249^a^	516.389*156.30^b^
60	3.251^#^	519.7*
100	3.250^#^	520.29*
200	3.246^#^	522.54*

^#^Measured value,

* Calculated value,

^a^Ref [65] Theor.,

^b^Ref. [66] Theor.

**Fig 9:**
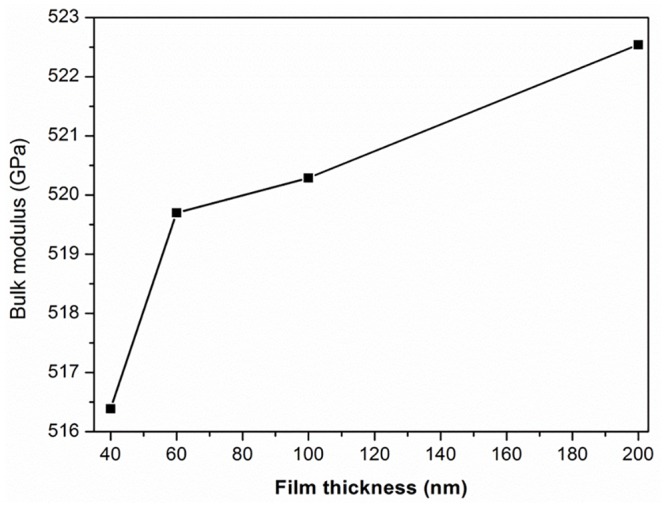
Fitted variation of bulk modulus of ZnO as a function of film thickness.

The dominant effect represents the degree of covalency that characterizes Phillips’ homopolar gap, ‘Eh’ [60], a qualitative concept, such as bulk modulus. These approaches were used in the present study because of the higher validity of our calculations.

$$B_{0}=\;\left[3000-100\lambda \right]{\left(\frac{a}{2}\right)}^{-3.5}$$ (9)

where ‘a’ is the lattice constant (in Å), and λ is an empirical parameter that accounts for the ionicity effect; λ = 0, 1, 2 for group IV, III–V, and II–VI semiconductors, respectively. In Table 3, the calculated bulk modulus values are compared with the theoretical [64,65] and experimental [66] values and fitted as shown in Fig 9. The bulk modulus of the ZnO nanostructure increases with the increasing current density, which attributes to the nanostructure. Previous studies showed similar chemical behavior to that observed from the experimental values displayed in Table 3.

**Table 3:** Calculated refractive indices of ZnO films at different current densities using models of Ravindra et al. [47], Herve and Vandamme [48], and Ghosh et al. [49] corresponding to the optical dielectric constant.

Thickness(nm)	Energy bandgap(eV)	Refractive index(n)	Optical dielectric constant (*ε*_∞_)
40	3.2823.22^a^3.25^b^	2.013^c^2.268^d^	4.052^c^5.14^d^
60	3.2793.22^a^3.25^b^	2.015^c^2.268^d^	4.060^c^5.14^d^
100	3.2773.22^a^3.25^b^	2.016^c^2.269^d^	4.064^c^5.15^d^
200	3.2683.22^a^3.25^b^	2.021^c^2.272^d^	4.084^c^5.16^d^

^a^ Kashif et al. (2012) Exp.;

^b^ Foo et al. (2014) Exp.;

^c^ Ravindra et al. (1979);

^d^ Herve and Vandamme (1995).

### Optical properties at varied pH

**Fig 10:**
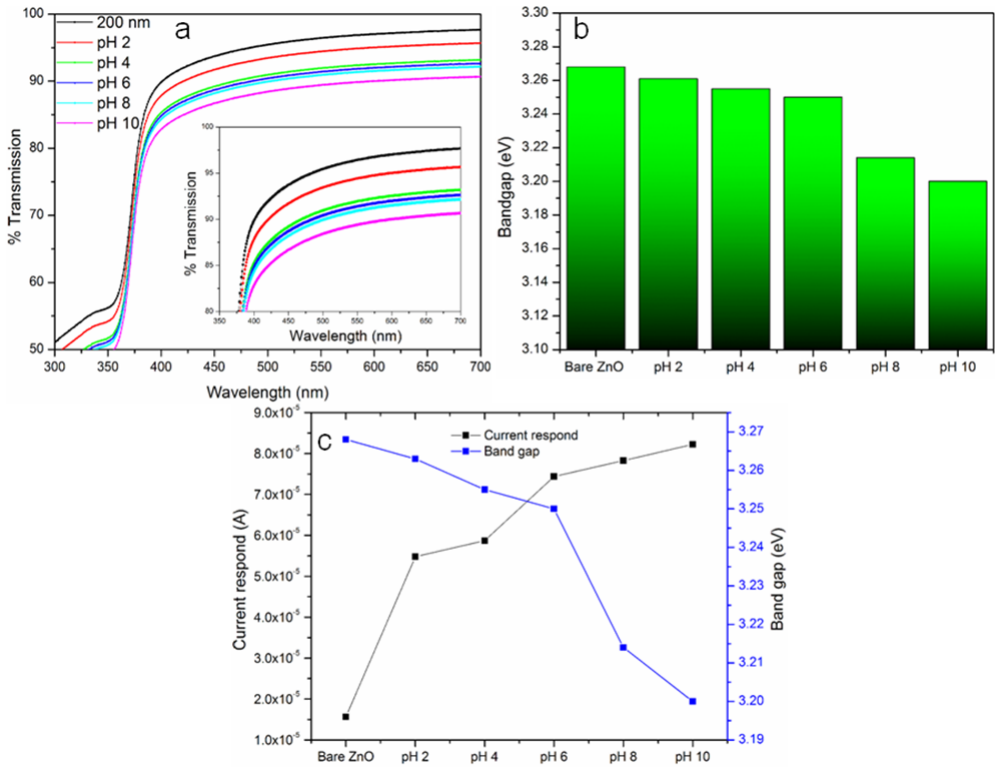
Optical behavior of membranous ZnO thin film in different pH condition. (a) Transmission spectra of ZnO thin film in solution of pH 2–10, (b) bandgap value of ZnO thin film in solution of pH 2–10 and (c) correlation between the current respond and bandgap of membranous ZnO thin film at pH 2–10.

Effects and changes in optical properties of the ZnO thin film were investigated under different pH conditions. Fig 10A illustrates the transmission spectrum of ZnO thin film with thickness of 200 nm, which was treated with different pH solutions (pH 2–10). It is noticeable that the intensity transmission spectrum deteriorates as the ZnO thin film is treated with increasing pH solutions.

As the pH values were increased, the transmission performances of ZnO thin film reduced. The transmission ability of the ZnO thin film reduced at basic conditions, might be due to formation of large particle at higher pH, which induces light scattering effect. The calculated bandgaps obtained for the ZnO thin film treated with various pH solutions are shown in Fig 10B. The bandgap values obtained under different conditions of pH 2 to 10 were 3.263 eV to 3.200 eV. According to Sivakumar et al. when the ZnO thin film was treated with buffer solutions of varied pH (pH 4–10) a remarkable reduction in bandgap value from 3.32 eV to 3.14 eV was observed [67]. Since there is no reduction in the grain size particle of ZnO thin film at low pH compared to as-synthesized ZnO, the etching effect at low pH condition did not exist. Fig 10C shows that at higher pH, the current response of the ZnO thin film was elevated and the band gap was reduced, which showed that the optical properties of ZnO thin film can be altered at different pH. Hence, it was evident that the changes in the optical properties of ZnO thin film at different pH can be applied for optical based pH sensor.

### Current to voltage (I-V) analysis on ZnO thickness with pH solutions

**Fig 11:**
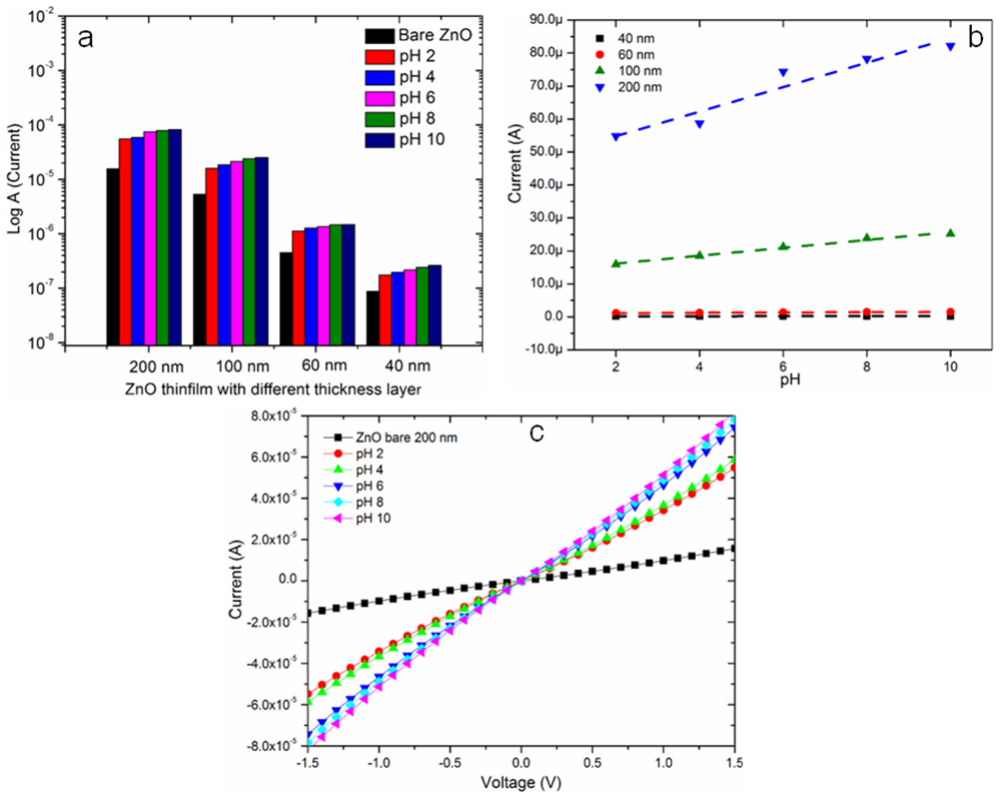
Electrical (I-V) behavior of developed sensor under different pH conditions. (a) Current response of ZnO thin films of different thicknesses for pH sensing (b) Sensitivity slope of pH sensor with different thickness layer of membranous ZnO thin film and (c) I-V result of optimized thickness (200 nm) of ZnO thin at different pH conditions are shown.

The current response of pH sensing is associated with ZnO thin films of different thicknesses, as obtained above (Fig 11A). ZnO thin films were coated with thicknesses of 40, 60, 100 and 200 nm on an IDE array with a p-type silicon substrate.

As depicted in Fig 11A, the current response of control ZnO IDE sensor increases with various thicknesses of membranous ZnO film. This is because, higher thickness of ZnO film leads to better growth of grain, which enables large grain sizes. This in turn causes bigger trap sites among the large grains that trap more charges and leads to increased conductivity. With higher thickness of thin film, deeper layer of atom form a stronger interatomic force, constructing a compact structure. Formation of compact structure with large thickness of ZnO thin film reduces structural defects and improves the crystallinity of the film which is consistent with the photoluminescence results [68].

All samples were treated with buffer solutions of different pH (2, 4, 6, 8 and 10). It is clearly illustrated in Fig 11A that samples of different thicknesses exhibit a similar increasing trend of current responses upon increasing the pH from 2 to 10. The current responses of ZnO thin film sensor showed tremendous conductivity increment when interfaced with aqueous acidic-basic pH, compared to the control IDE sensor. At lower pH (pH <7), the conductivity behavior of the thin film was higher due to diffusion of hydrogen (H^+^) on the surface of ZnO. Generally, hydrogen behaves as a shallow donor which contributes to the improvement of conductivity performances of the host ZnO thin film by donating the electron to the conduction band of the ZnO.

The following chemical reaction represents the reaction of ZnO in an acidic solution:

$$ZnO+\;H^{+}↔Zn{\left(OH_{2}\right)}^{+}$$ (10)

In a highly acidic solution, hydroxyl (OH) groups on the ZnO adsorb protons from hydrogen (H^+^) produces positively charged surface. In a basic solution, hydroxyl groups on ZnO lose proton from OH^-^ and forms negatively charged surface [69]. Hence, as the pH of the solution increases, electron mobility on the surface of membranous ZnO thin film increases, which causes an increment in current response of the ZnO IDE pH sensor.

$$ZnO+\;OH^{-}↔Zn{\left(O\right)}^{-}+\;H_{2}O$$ (11)

Fig 11B shows the slope of the current response for the entire IDE sensor associated with different thickness layers of ZnO, increases with increasing film thicknesses. The slope values obtained for thicknesses from 40 nm to 200 nm were 1.097 x 10^−8^ to 3.72 x 10^−6^ as shown in Table 4. Hence the conductivity of the IDE sensor with increased thickness of ZnO film was improved due to larger grain size which contributes to large trap sites.

**Table 4:** Linear regression, R^2^, slope of calibration plot, and sensitivity of ZnO thin films of different thicknesses tested with pH 2–10.

ZnO thin film Thickness (nm)	Linear Regression, R^2^	Slope of calibration plot, m(μAmM^-1^)	Sensitivity,μAmM^-1^cm^-2^
40	0.9968	1 x 10^−8^	1.11 μA
60	0.9456	4 x 10^−8^	4.44 μA
100	0.9878	1 x 10^−6^	111 μA
200	0.9304	4 x 10^−6^	444 μA

### Sensitivity and stability of optimized ZnO IDE sensor

Fig 11C represents the I-V result from the hetero-junction of the ZnO thin film (200 nm) that was coated on an IDE (interdigitated electrode), which can be applied for bio-recognition. Fig 11C clearly illustrates that the ZnO thin film showed ohmic contact with the potential voltage supply in the range of -1.5 to 1.5 V. With these optimized characteristics applied as a transducer, we performed experiments at different pHs. When the IDE sensor was treated at an extremely acidic solution (pH 2) exhibited a low current with a value of 5.48 x 10^−5^ A, and this value was increased as the pH value of the solution became basic. The highest current, exhibited at pH 10, was 8.22 x 10^-5^A. From this result, it is clear that the ZnO thin film can be utilized as a pH sensing membrane that responds to the concentration of H^+^ in an acidic solution and of OH^-^ in a basic solution.

Further investigation on the sensitivity of the pH response was conducted on ZnO thin films of different thicknesses (Table 4). The range of linear regression, R^2^, values obtained was from 0.9304 to 0.9968. The sensitivity was calculated using the following formula [70]:

$$\text{Sensitivity}=\frac{\text{Slope of calibration plot,m }\left(\mu \text{A mM-1}\right)}{\text{Active Surface Area,A }\left(\text{cm2}\right)}$$ (12)

**Fig 12:**
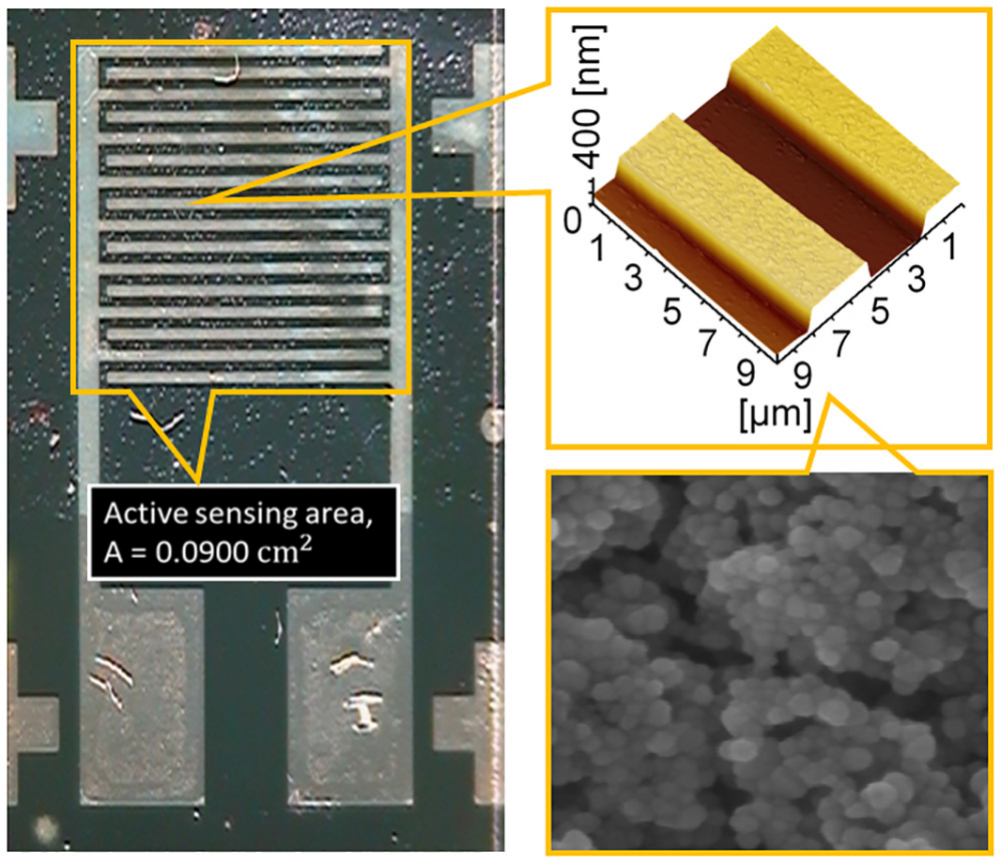
Overall fabrication of ultra-sensitive silver interdigitated electrode (IDE) for optoelectronic and pH sensing mechanism by simple and economical lithography route. Fabricated IDE array was coated with a ZnO thin film.

**Fig 13:**
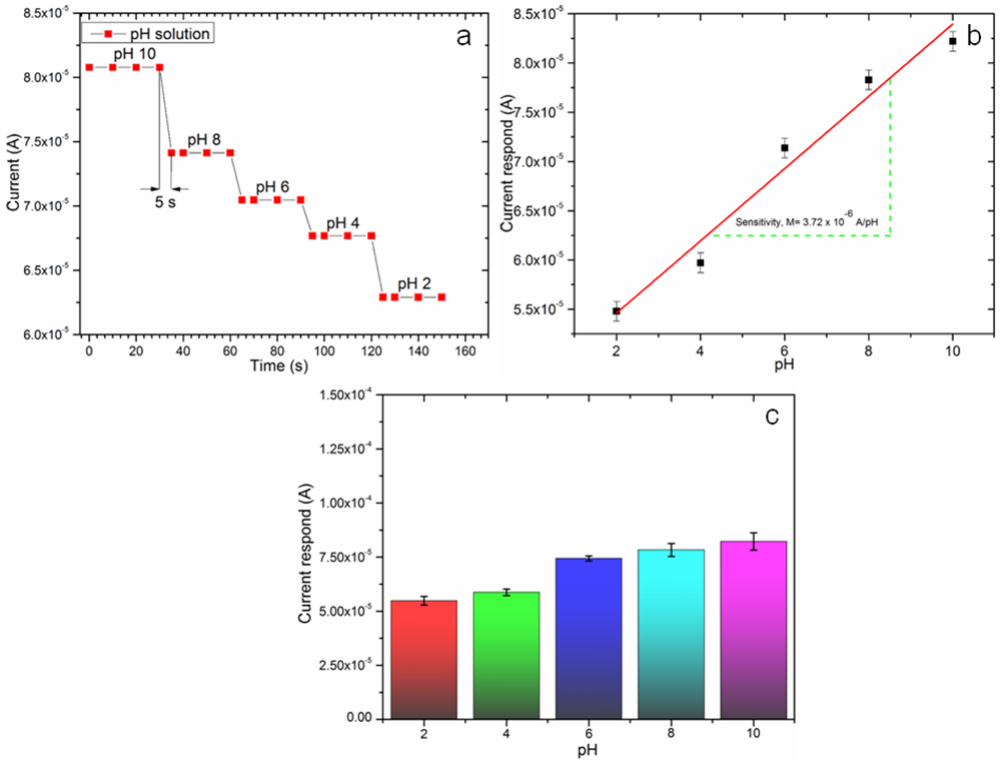
Analytical performance of the optimized ZnO thin film pH sensor. (a) Current-time (s) response of the IDE ZnO thin film pH sensor, (b) Sensitivity slope per pH unit of IDE ZnO thin film sensor, and (c) Stability performance of sensor at different pH condition.

The highest sensitivity was obtained for the ZnO thin film with the greatest thickness (200 nm), 444 μAmM^-1^cm^-2^ on the geometrically active sensing area, A = 0.00900 cm^2^ (Fig 12). Thus, the enhanced sensitivity of the pH sensor is associated with a higher thickness ZnO thin film. From these results, it can be concluded that a higher thickness thin film has a larger active surface area due to its superior crystalline quality with low crystal defect which is good agreement with Fig 8. The current response-time of the device at different pH was measured, which shows 96–98% of steady-state current within 5 seconds (Fig 13A). The sensitivity of the developed device per pH unit obtained was 3.72 μA/pH, which is illustrated in Fig 13B. The electrical stability of the fabricated device at different pH was also measured as depicted in Fig 12C. As shown in Fig 13C, the stability performances of the device is optimum at pH 6 and the stability performance deviates at higher pH (pH 8–10) and at low pH (pH 2–4). S1 Fig shows that the current response of the device treated prior with acidic pH condition is similar after the device was washed with DI water which shows that the etching effect on the membranous ZnO thin film did not exist and the device performs based on pure ionic condition [71,72].

Table 5 illustrates the comparison of developed pH sensors with various types of electrode systems. Based on the data obtained in Table 5, it is clearly shown that our present work have superior performances compared to previous works. The developed IDE comprises fast responses compared to other electrode systems, which only require 5 seconds suitable for wide range pH. One of the promising aspects of our current work is that, ultra-low volume of pH solution is sufficient for the developed IDE device. Hence, based on the morphological, optical and electrical characterizations of the thickness of ZnO thin film, highest thickness of the membranous ZnO thin film was justified as the most optimum sensor performances for pH sensing application.

**Table 5:** Comparison of developed pH sensors with various types of sensing electrode system.

Types of sensing electrode	Sensing membrane platform	pH range	Sensitivity per pH unit	Response time	Liquid volume	Sensing film thickness	Reference
Extended gate thin film transistor (EGTFT)	Nanoporous ZnO	1–12	59 mV/pH	-	Immersed in solution	700 nm	[2]
EIS sensor	ZnO thin film	2–12	42.54 mV/pH	2.40 mV/h	Immersed in solution	50 nm	[73]
Microcantilever	Copolymetric hydrogels		20.3 μm (1nm/5 x 10^−5^ Δ pH)	30 min	Immersed in solution	-	[74]
Ion-Sensitive field effect transistor (ISFET)	ZnO thin film	4–12	48.27mV/pH	Less than 12 s	Immersed in solution	80 nm	[75]
Interdigitated electrode (IDE) Array	ZnO thin film	2–10	3.72 μA/pH	5 s	2mL	40–200 nm	Present work
Intracellular sensor	ZnO Nanorods	4–11	51.88 mV/pH	5 min	Immersed in solution	-	[76]

## Conclusions

ZnO thin films of different thicknesses were deposited on silicon and glass substrates. An increase in the film thickness greatly influences the crystallinity, surface morphology, and optoelectronic properties of the thin film. AFM data show that the RMS surface roughness of the thin film decreases from 23.00 to 3.58 nm with the increasing film thicknesses. The optical transmission of the ZnO thin film was highest for the glass substrate coated with the ZnO thin film of the lowest thickness. The band gap energy of the thin film can also be altered by adjusting its thickness. The obtained results for the refractive index, optoelectric constant, and bulk modulus of ZnO thin films with thicknesses from 40 to 200 nm were in agreement with theoretical observations. The bandgap value of ZnO thin film (200 nm) reduces with increasing pH conditions (pH 2–10). Therefore, this study could contribute to optimization procedures for synthesizing highly crystallined ZnO thin film. The optimized ZnO thin film is suited for pH sensing, showing a promising stability under varied pH conditions from more acidic to basic, with a highly sensitive response of 444 μAmM^-1^cm^-2^ with a linear regression of R^2^ = 0.9304. The sensitivity performance of the device for per pH unit is 3.72μA (3.72μA/pH).

## Supporting Information

S1 Fig Analytical performance of the optimized ZnO thin film pH sensor. Variations in current response prior and after treated with pH solution.
(PPTX)
