# Supplementary figures and images for: Low Temperature Annealed Zinc Oxide Nanostructured Thin Film-Based Transducers: Characterization for Sensing Applications
Source: PLoS One. 2015 Jul 13;10(7):e0132755. doi: 10.1371/journal.pone.0132755 (PMC4500498; doi:10.1371/journal.pone.0132755)

## Slide 1
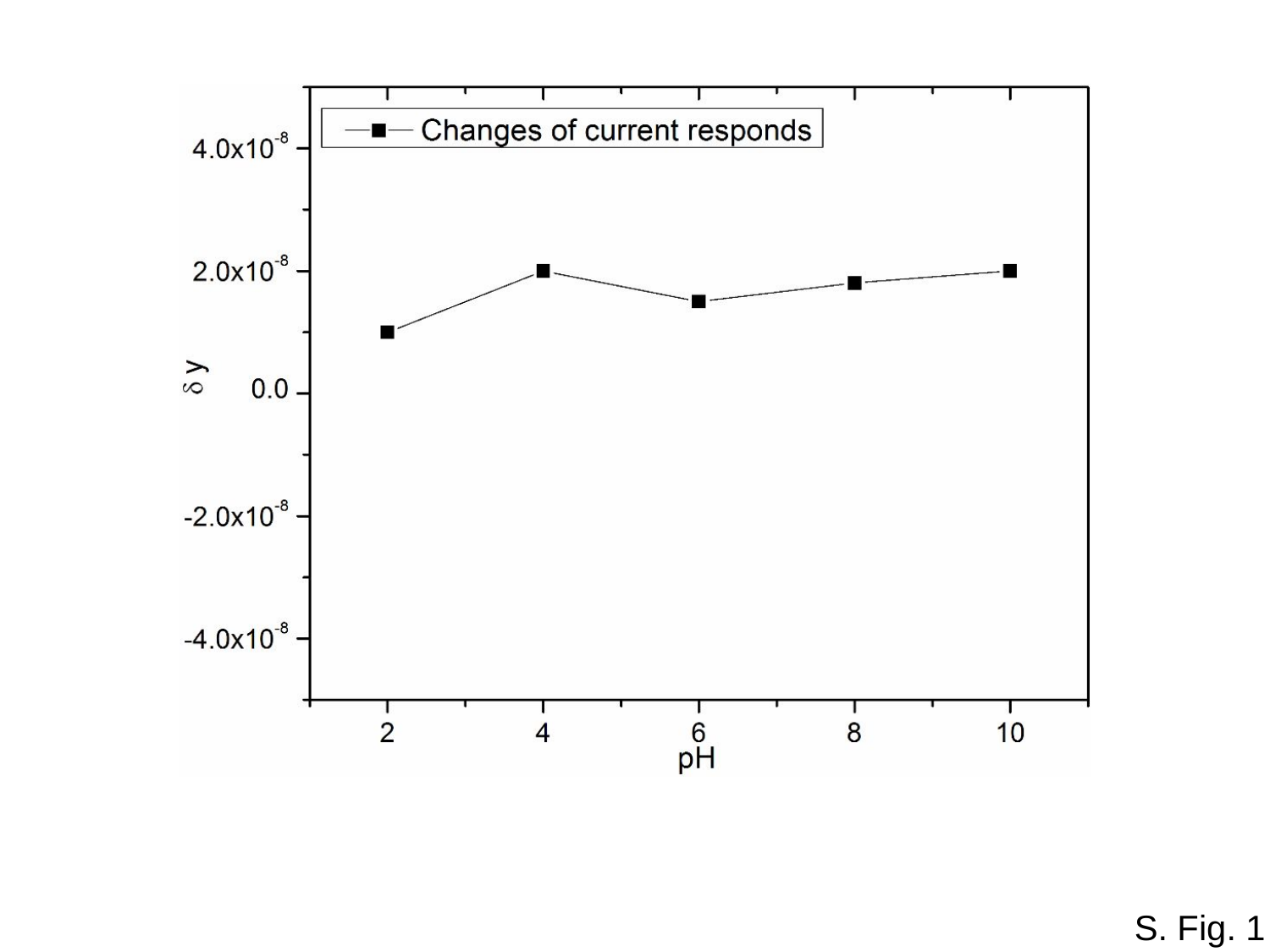

S. Fig. 1

Supplement: S1 Fig — Variations in current response prior and after treated with pH solution. (PPTX) [file pone.0132755.s001.pptx]
